# A zinc-chelating cyclic alkyl polyamine compound is efficient and safe in a murine model of multidrug-resistant *Candida auris* infection

**DOI:** 10.1128/aac.00856-25

**Published:** 2025-09-26

**Authors:** Takayuki Shinohara, Akira Wada, Masahiro Abe, Sayoko Oiki, Ami Koizumi, Amato Otani, Harutaka Katano, Yoshitsugu Miyazaki

**Affiliations:** 1 Department of Fungal Infection, National Institute of Infectious Diseases, Japan Institute for Health Security 739298 , Tokyo, Japan; 2 Center for Integrative Medical Sciences, RIKEN, Kanagawa, Japan; 3 Department of Infectious Disease Pathology, National Institute of Infectious Diseases, Japan Institute for Health Security 739298 , Tokyo, Japan; 4 Leprosy Research Center, National Institute of Infectious Diseases, Japan Institute for Health Security 739298 , Tokyo, Japan; University of Iowa, Iowa City, Iowa, USA

**Keywords:** *Candida auris*, multidrug-resistant fungi, disseminated candidiasis, novel antifungal agents, zinc chelation, metal homeostasis

## Abstract

*Candida auris* is an emerging multidrug-resistant fungal pathogen associated with severe nosocomial outbreaks and high mortality rates worldwide. The increasing incidence of antifungal resistance underscores the urgent need for agents with novel mechanisms of action. APC6 is a zinc-chelating cyclic alkyl polyamine compound that selectively disrupts zinc homeostasis in fungal cells. We have previously reported that APC6 has antifungal activity against *Candida* spp., including *Candida auris*, and low cytotoxicity to human cells. In this study, we evaluated the *in vivo* efficacy and safety of APC6 using a neutropenic murine model of disseminated *C. auris* infection. APC6 significantly improved survival and reduced fungal burden in the liver, kidneys, and brain. At a therapeutic dose of 15 mg/kg, APC6 had similar or superior antifungal activity to that of amphotericin B. Histopathological analysis revealed a decreased number of fungal microabscesses in APC6-treated tissues. No significant adverse effects were observed following 28-day repeated intraperitoneal administration, and the Ames assay revealed no mutagenic activity. To our knowledge, this is the first study to demonstrate that a zinc-chelating compound can improve survival and reduce organ fungal burden in a mammalian model of drug-resistant *C. auris* infection. These results highlight APC6 as a promising lead compound targeting fungal zinc homeostasis and support its further development as a novel antifungal agent.

## INTRODUCTION

The global burden of invasive fungal infections continues to rise, presenting an urgent public health threat (1). This is particularly evident in immunocompromised patients, such as those undergoing chemotherapy, organ transplantation, or treatment with broad-spectrum antibiotics, who are increasingly susceptible to systemic fungal pathogens including *Candida*, *Aspergillus*, and *Cryptococcus* spp. (2, 3). *Candida auris* was first identified in 2009 and has rapidly emerged as a multidrug-resistant yeast responsible for nosocomial outbreaks across more than 50 countries, often resulting in bloodstream infections with mortality rates exceeding 30% (4). *C. auris* can be transmitted between individuals, persists in the environment, and presents substantial diagnostic challenges. One major concern is its poor susceptibility to antifungal agents (4). Over 90% of clinical isolates are resistant to fluconazole, and resistance to voriconazole, amphotericin B, and echinocandins is increasing (5). Some isolates are resistant to all three major antifungal drug classes—namely, polyenes, azoles, and echinocandins (4)—limiting treatment options and complicating clinical management. In recognition of its clinical impact, the World Health Organization listed *C. auris* as a “critical priority” fungal pathogen in 2022 (6).

Biological similarities between fungi and humans hamper the identification of pathogen-specific drug targets and increase the risk of toxicity to the host (7). Currently approved antifungal drugs primarily target ergosterol or β-glucan biosynthesis, whereas potential drugs with novel mechanisms of action, including interference with metal homeostasis, protein acylation, or mitochondrial function, are being investigated (8–10). Identification of novel antifungal mechanisms remains a critical unmet need in the field.

An emerging therapeutic strategy involves perturbing fungal metal homeostasis, particularly by targeting zinc and iron acquisition systems, which are essential for pathogenicity and cellular viability (11). Zinc chelation as an antifungal strategy aligns with the concept of nutritional immunity (12), which is a host’s defense mechanism wherein the availability of essential nutrients—particularly metal ions—is restricted to inhibit pathogen growth during infection (13). Fungal and bacterial pathogens require metal cations for survival and proliferation within the host and have evolved specialized mechanisms to acquire these metals (14). Notably, the concentration of free Zn²^+^ in human serum (ca. 0.08 µM) is about 150 times lower than the minimum zinc concentration required for optimal growth of *Candida albicans* or *Aspergillus fumigatus* (15, 16). Therefore, pathogenic fungi must efficiently acquire zinc to survive within the host. Specifically, mammalian immune cells restrict fungal access to zinc through several zinc-binding proteins, including calprotectin, psoriasin, and calgranulin, thereby limiting fungal proliferation (17). Inspired by nutritional immunity, we hypothesized that mimicking the host’s zinc sequestration mechanism could offer a novel antifungal therapeutic approach.

Recent preclinical studies have highlighted the potential of synthetic metal chelators as antifungal agents (15–18). Compounds ZAC307 and ZAC989 inhibit zinc utilization in *C. albicans* and *A. fumigatus*, and they significantly reduce fungal growth in both *in vitro* and murine models (19). Thiosemicarbazone derivatives such as NSC319726 and 19ak disrupt mitochondrial respiration and ribosome biogenesis through iron and zinc chelation, respectively, and have broad-spectrum antifungal activity (17, 18). NSC319726 is efficient *in vivo* against *C. auris* in *Galleria mellonella* larvae (20). These studies suggest the feasibility of targeting metal acquisition pathways as a novel antifungal strategy.

**Fig 1:**
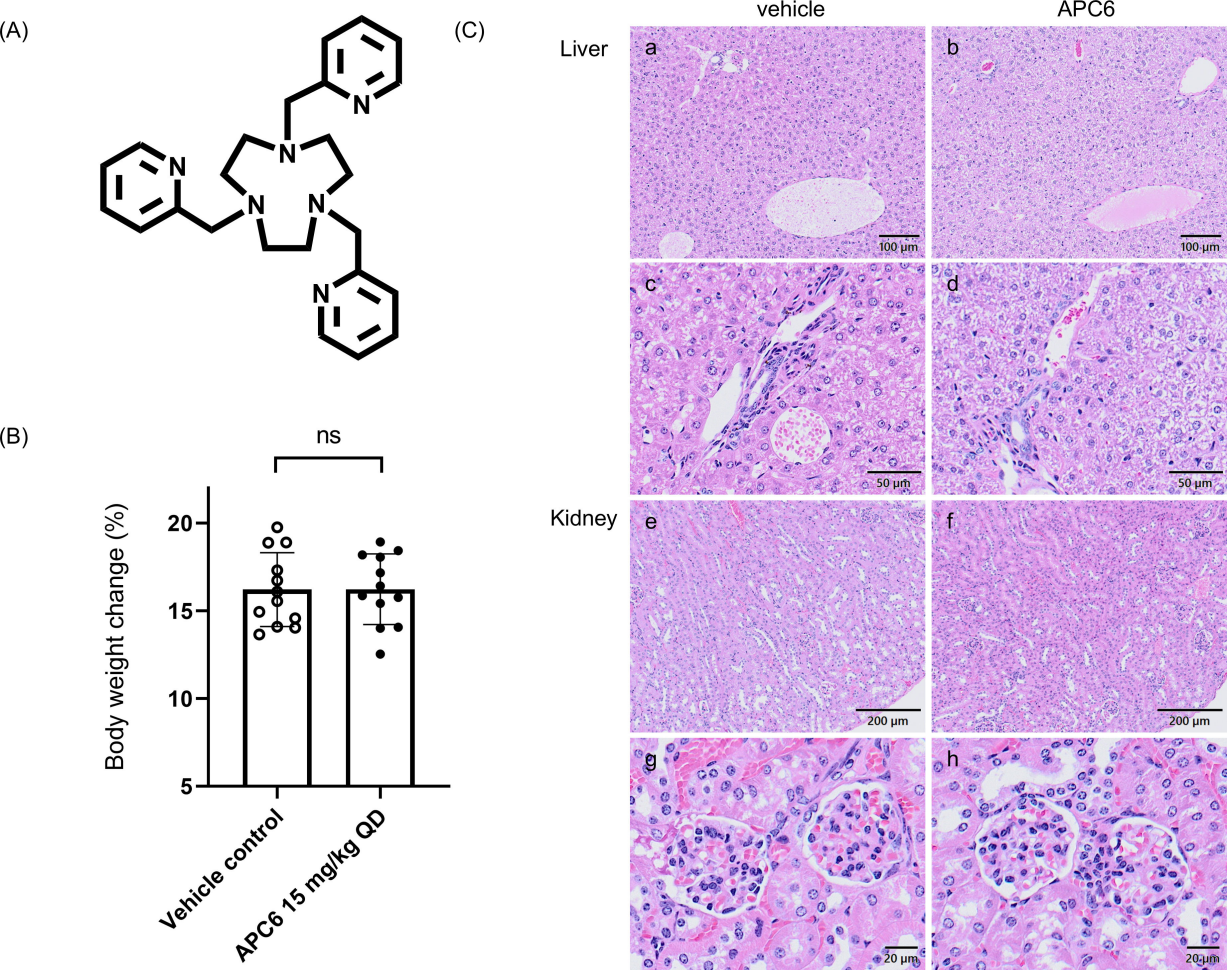
(**A**) Chemical structure of APC6. (**B**) Body weight changes in mice receiving daily intraperitoneal injections of APC6 for 28 consecutive days (*n* = 12 per group). Data are the mean ± standard error of the mean from three independent experiments. ns, no significant difference (two-tailed unpaired Student’s *t*-test); QD (quaque die), once daily. (**C**) Representative histopathological sections of liver and kidney tissues collected after 28 consecutive days of APC6 administration, stained with hematoxylin and eosin. (a and b) Liver: the portal vein and Glisson’s sheath; (c and d) high-magnification views of Glisson’s sheath; (e and f) kidneys: renal cortex; (g and h) representative glomerular structures.

Our group has previously reported the *in vitro* antifungal activity of the cyclic alkyl polyamine compound (APC) APC6 (18). APCs, which consist of ethylenediamine units, can chelate divalent metal ions. In particular, their cyclic rigid structures allow the stable formation of metal complexes. Introduction of pyridylmethyl groups at the nitrogen atoms further enhances their metal-binding affinities. The positively charged APCs are likely to interact with negatively charged fungal membranes. Based on prior knowledge of coordination chemistry and mycology, we synthesized a series of linear and cyclic APCs and screened them for antifungal activity (18), leading to the identification of APC6 (Fig. 1A). APC6 is a high-affinity chelator for zinc and selectively perturbs intracellular zinc homeostasis in fungal cells. *In vitro*, APC6 forms a stable 1:1 complex with Zn²^+^, with a logK of 17.25—exceeding that of EDTA—as revealed by electrospray ionization mass spectrometry (18). The addition of Zn²^+^ to culture media reverses the antifungal activity of APC6; intracellular zinc levels in *Candida* cells decrease significantly in an APC6 concentration-dependent manner, as measured by the zinc-specific fluorescent probe. Comprehensive transcriptomic and reverse transcription quantitative PCR analyses have shown that APC6 induces the expression of zinc transporter and scavenger genes but not those related to other metal ions, suggesting selective zinc deprivation as its principal mode of action (18).

APC6 selectively perturbs intracellular zinc homeostasis in fungal cells and effectively inhibits the growth of various *Candida* spp., including multidrug-resistant *C. auris* (18). Therapeutic efficacy of APC6 has been demonstrated in *G. mellonella* larvae, but its effectiveness in mammalian hosts is unknown.

In this study, we evaluated the *in vivo* efficacy and safety of APC6 using a neutropenic murine model of disseminated *C. auris* infection, employing two multidrug-resistant clinical isolates from clades I and III. To our knowledge, this is the first report that a zinc-chelating compound significantly improves survival and reduces organ fungal burden in a mammal infected with *C. auris*. This study provides *in vivo* evidence that targeting fungal zinc homeostasis may represent a viable antifungal strategy.

## RESULTS

### APC6 has a favorable safety profile at 15 mg/kg in mice

We first evaluated the safety of APC6 (Fig. 1A) through a single-dose de-escalation study and a 28-day repeated-dose toxicity study in mice. In the single-dose study, administration of 45 mg/kg resulted in mortality within 1 day and 30 mg/kg induced transient tremors, whereas 15 mg/kg was well tolerated without observable clinical signs and was therefore designated as the maximum tolerated dose. In the repeated-dose study, mice were intraperitoneally injected with APC6 at 15 mg/kg/day for 28 consecutive days, in accordance with Organisation for Economic Co-operation and Development Guideline for the Testing of Chemicals, Repeated Dose 28-Day Oral Toxicity Study in Rodents and previous studies (21, 22). Throughout the study period, no significant clinical abnormalities were observed. All APC6-treated and control mice maintained a grade 1 activity level throughout the study period. Body weight changes did not differ significantly between the APC6-treated and vehicle control groups (Fig. 1B). Histopathological analysis of the liver and kidneys at day 28 revealed no gross morphological alterations, inflammation, or fibrosis in the APC6-treated mice compared with the controls (Fig. 1C). In the liver, Glisson’s sheath—comprising the portal vein, hepatic artery, and bile duct—was clearly observed without any signs of inflammatory cell infiltration or fibrosis. The central vein, hepatic lobules, and hepatocytes appeared normal, with no evidence of degeneration or inflammation. The glomerular architecture of the renal cortex was well preserved, with no signs of glomerular atrophy, mesangial proliferation, or interstitial inflammation. No significant microscopic differences were observed in either organ between the APC6-treated and vehicle control groups.

### APC6 improves survival in a murine model of disseminated *C. auris* infection

**Fig 2:**
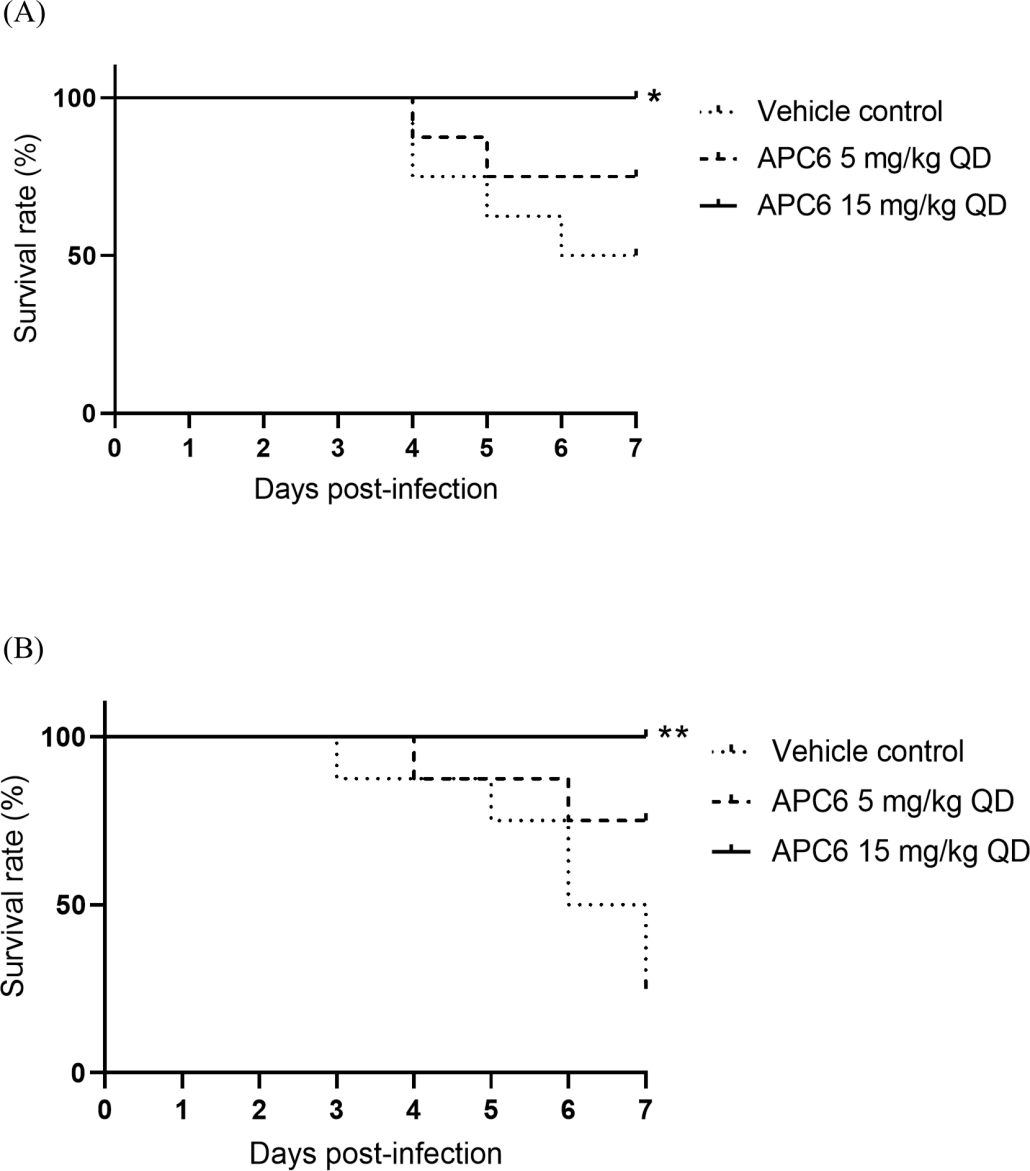
Kaplan–Meier survival curves of mice infected with *C. auris* strains (**A**) AR 0384 and (**B**) AR 0389. Mice were injected intraperitoneally (*n* = 8 per group) with APC6 at the indicated doses, or with vehicle. Survival was monitored for 7 days post-infection. Statistical significance was assessed by the log-rank test. *P < 0.05, **P < 0.01 vs control. QD (quaque die), once daily.

We assessed the therapeutic efficacy of APC6 in neutropenic mice infected intravenously with *C. auris* after immunosuppression by cyclophosphamide. We used *C. auris* strains AR 0384 (clade III) and AR 0389 (clade I), both of which are highly resistant to fluconazole; AR 0384 is additionally resistant to echinocandins—particularly caspofungin—and AR 0389 to amphotericin B, as reported by the Centers for Disease Control and Prevention (CDC) (Table S1). Mice were challenged intravenously with either 5 × 10⁷ colony-forming units (CFU) of AR 0384 or 3 × 10⁷ CFU of AR 0389 per mouse. APC6 was administered intraperitoneally once daily at a dose of 5 or 15 mg/kg, starting at 24 h before infection and continuing thereafter; this regimen was based on a previous *in vivo* evaluation of zinc-chelating compounds (19). Survival was monitored for 7 days post-infection. In the AR 0384-infected cohort, the survival rate was 50% in the vehicle control group, 75% in the APC6 5 mg/kg group, and 100% in the APC6 15 mg/kg group (Fig. 2A). In the AR 0389-infected cohort, the survival rate was 25% in the vehicle group, 75% in the 5 mg/kg group, and 100% in the 15 mg/kg group (Fig. 2B).

To further investigate whether the *in vivo* antifungal activity of APC6 is mediated by zinc chelation and to assess its long-term efficacy, we conducted a 21-day survival experiment using *C. auris* strain AR 0384 with or without co-administration of zinc. Zinc supplementation markedly diminished the protection conferred by APC6 (Fig. S1), supporting zinc chelation as a key mechanism of APC6 action *in vivo*.

These findings indicate that APC6 significantly improves survival in a murine model of disseminated *C. auris* infection.

### APC6 reduces fungal burden in the liver, kidneys, and brain

**Fig 3:**
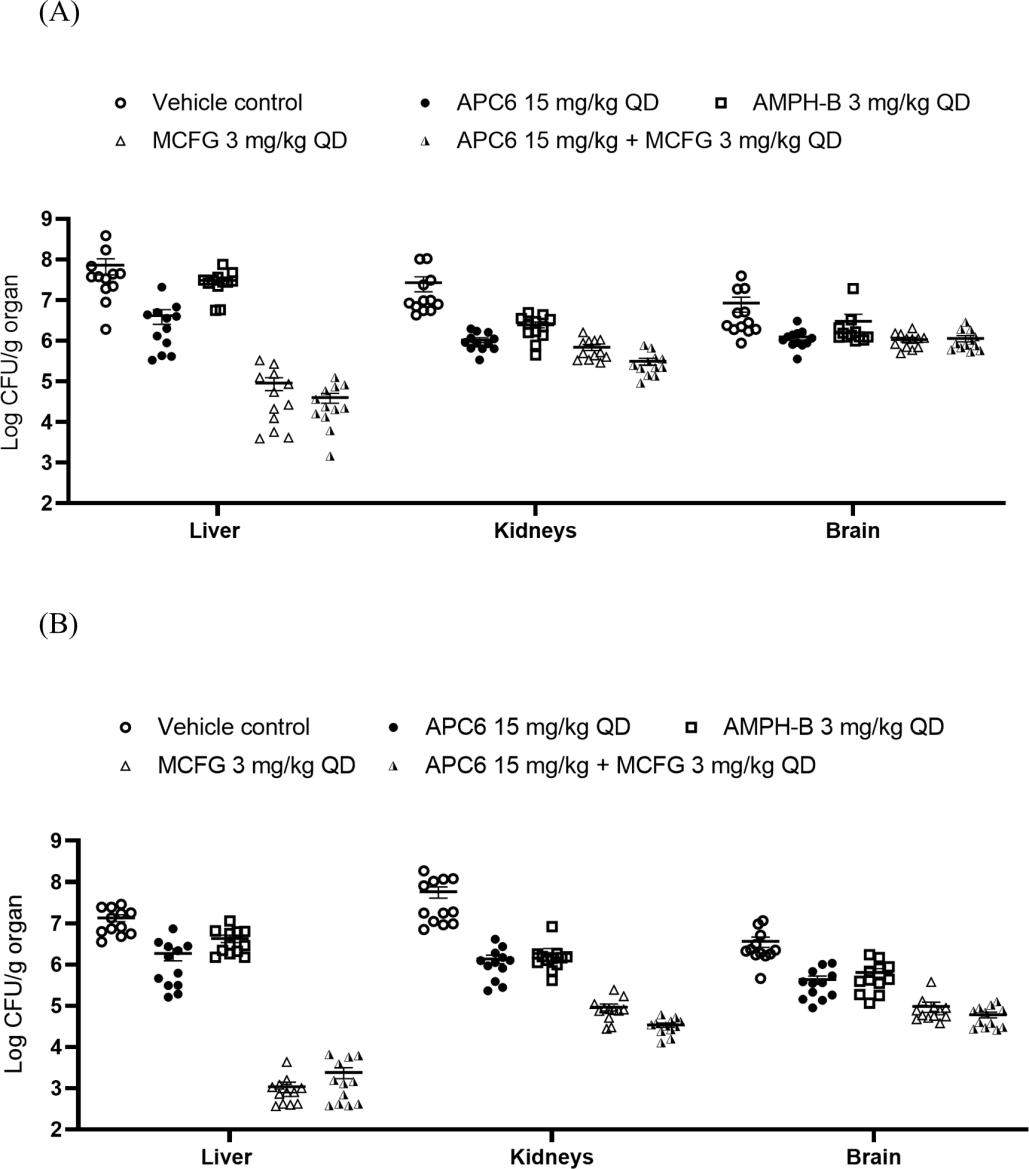
Fungal burden in the liver, kidneys, and brain of mice infected with *C. auris* strains (**A**) AR 0384 and (**B**) AR 0389. Mice were injected intraperitoneally (*n* = 12 per group) with APC6, AMPH-B, MCFG, APC6 combined with MCFG, or vehicle control. Mice were euthanized on day 3 post-infection, and fungal burden was determined by plating organ homogenates. Data are the mean ± standard error of the mean from at least three independent experiments. Statistical significance was assessed by one-way analysis of variance followed by Tukey’s multiple comparison test. Statistical comparisons are summarized in Table 1 (AR 0384) and Table 2 (AR 0389). AMPH-B, amphotericin B; MCFG, micafungin; QD (quaque die), once daily.

To evaluate the effect of APC6 on fungal burden, we quantified CFU in the liver, kidneys, and brain at day 3 post-infection. In the AR 0384-infected cohort, APC6 at 15 mg/kg significantly reduced the fungal burden in comparison with the vehicle control group: the mean reductions were 1.226 log_10_ CFU/g in the liver, 1.170 log_10_ CFU/g in the kidneys, and 0.584 log_₁₀_ CFU/g in the brain (Fig. 3A; Table 1). Among monotherapies, micafungin achieved the greatest reduction in fungal burden. Combination therapy with APC6 and micafungin did not show additive effects compared with micafungin alone. In the AR 0389-infected cohort, APC6 at 15 mg/kg also significantly reduced fungal burden in comparison with the vehicle group: by 1.056 log_10_ CFU/g in the liver, 1.498 log_10_ CFU/g in the kidneys, and 0.902 log_10_ CFU/g in the brain (Fig. 3B; Table 2). These results demonstrate that APC6 effectively reduces the fungal burden in multiple organs in this murine model.

**TABLE 1:** Statistical summary of between-group comparisons of average log_10_ CFU/g of organs in mice infected with *C. auris* AR 0384

Group 1	Group 2	Mean difference	95% CI^*a*^	Adjusted *P* value
Liver				
APC6 15 mg/kg	Vehicle control	−1.226	−1.862 to −0.589	<0.0001
AMPH-B 3 mg/kg	Vehicle control	−0.147	−0.783 to 0.490	0.966
MCFG 3 mg/kg	Vehicle control	−2.983	−3.619 to −2.346	<0.0001
APC6 15 mg/kg + MCFG 3 mg/kg	Vehicle control	−3.165	−3.802 to −2.528	<0.0001
APC6 15 mg/kg + MCFG 3 mg/kg	APC6 15 mg/kg	−1.939	−2.576 to −1.303	<0.0001
APC6 15 mg/kg + MCFG 3 mg/kg	AMPH-B 3 mg/kg	−3.018	−3.655 to −2.382	<0.0001
APC6 15 mg/kg + MCFG 3 mg/kg	MCFG 3 mg/kg	−0.182	−0.819 to 0.454	0.9274
MCFG 3 mg/kg	APC6 15 mg/kg	−1.757	−2.394 to −1.120	<0.0001
MCFG 3 mg/kg	AMPH-B 3 mg/kg	−2.836	−3.473 to −2.200	<0.0001
APC6 15 mg/kg	AMPH-B 3 mg/kg	−1.079	−1.716 to −0.443	0.0001
Kidneys				
APC6 15 mg/kg	Vehicle control	−1.170	−1.538 to −0.803	<0.0001
AMPH-B 3 mg/kg	Vehicle control	−0.828	−1.196 to −0.461	<0.0001
MCFG 3 mg/kg	Vehicle control	−1.363	−1.731 to −0.996	<0.0001
APC6 15 mg/kg + MCFG 3 mg/kg	Vehicle control	−1.726	−2.093 to −1.360	<0.0001
APC6 15 mg/kg + MCFG 3 mg/kg	APC6 15 mg/kg	−0.556	−0.923 to −0.189	0.0007
APC6 15 mg/kg + MCFG 3 mg/kg	AMPH-B 3 mg/kg	−0.898	−1.265 to −0.531	<0.0001
APC6 15 mg/kg + MCFG 3 mg/kg	MCFG 3 mg/kg	−0.363	−0.730 to 0.004	0.0543
MCFG 3 mg/kg	APC6 15 mg/kg	−0.193	−0.560 to 0.174	0.5789
MCFG 3 mg/kg	AMPH-B 3 mg/kg	−0.535	−0.902 to −0.168	0.0012
APC6 15 mg/kg	AMPH-B 3 mg/kg	−0.342	−0.709 to 0.025	0.0793
Brain				
APC6 15 mg/kg	Vehicle control	−0.584	−0.957 to −0.211	0.0005
AMPH-B 3 mg/kg	Vehicle control	−0.369	−0.743 to 0.004	0.0542
MCFG 3 mg/kg	Vehicle control	−0.627	−1.001 to −0.254	0.0001
APC6 15 mg/kg + MCFG 3 mg/kg	Vehicle control	−0.629	−1.003 to −0.256	0.0001
APC6 15 mg/kg + MCFG 3 mg/kg	APC6 15 mg/kg	−0.045	−0.419 to 0.328	0.997
APC6 15 mg/kg + MCFG 3 mg/kg	AMPH-B 3 mg/kg	−0.260	−0.633 to 0.114	0.2976
APC6 15 mg/kg + MCFG 3 mg/kg	MCFG 3 mg/kg	−0.002	−0.375 to 0.372	>0.9999
MCFG 3 mg/kg	APC6 15 mg/kg	−0.043	−0.417 to 0.330	0.9975
MCFG 3 mg/kg	AMPH-B 3 mg/kg	−0.258	−0.632 to 0.115	0.3043
APC6 15 mg/kg	AMPH-B 3 mg/kg	−0.215	−0.588 to 0.159	0.4898

^*a*^ CI, confidence interval.

**TABLE 2:** Statistical summary of between-group comparisons of average log_10_ CFU/g of organs in mice infected with *C. auris* AR 0389

Group 1	Group 2	Mean difference	95% CI	Adjusted *P* value
Liver				
APC6 15 mg/kg	Vehicle control	−1.056	−1.527 to −0.584	<0.0001
AMPH-B 3 mg/kg	Vehicle control	−0.499	−0.970 to −0.027	0.0333
MCFG 3 mg/kg	Vehicle control	−4.117	−4.589 to −3.646	<0.0001
APC6 15 mg/kg + MCFG 3 mg/kg	Vehicle control	−3.898	−4.369 to −3.426	<0.0001
APC6 15 mg/kg + MCFG 3 mg/kg	APC6 15 mg/kg	−2.842	−3.314 to −2.371	<0.0001
APC6 15 mg/kg + MCFG 3 mg/kg	AMPH-B 3 mg/kg	−3.399	−3.871 to −2.928	<0.0001
APC6 15 mg/kg + MCFG 3 mg/kg	MCFG 3 mg/kg	0.220	−0.252 to 0.691	0.6843
MCFG 3 mg/kg	APC6 15 mg/kg	−3.062	−3.533 to −2.590	<0.0001
MCFG 3 mg/kg	AMPH-B 3 mg/kg	−3.619	−4.090 to −3.147	<0.0001
APC6 15 mg/kg	AMPH-B 3 mg/kg	−0.557	−1.029 to −0.086	0.0129
Kidney				
APC6 15 mg/kg	Vehicle control	−1.498	−1.905 to −1.090	<0.0001
AMPH-B 3 mg/kg	Vehicle control	−1.354	−1.761 to −0.946	<0.0001
MCFG 3 mg/kg	Vehicle control	−2.607	−3.015 to −2.200	<0.0001
APC6 15 mg/kg + MCFG 3 mg/kg	Vehicle control	−3.003	−3.411 to −2.596	<0.0001
APC6 15 mg/kg + MCFG 3 mg/kg	APC6 15 mg/kg	−1.506	−1.913 to −1.098	<0.0001
APC6 15 mg/kg + MCFG 3 mg/kg	AMPH-B 3 mg/kg	−1.650	−2.057 to −1.242	<0.0001
APC6 15 mg/kg + MCFG 3 mg/kg	MCFG 3 mg/kg	−0.396	−0.804 to 0.012	0.0608
MCFG 3 mg/kg	APC6 15 mg/kg	−1.110	−1.517 to −0.702	<0.0001
MCFG 3 mg/kg	AMPH-B 3 mg/kg	−1.254	−1.661 to −0.846	<0.0001
APC6 15 mg/kg	AMPH-B 3 mg/kg	−0.144	−0.552 to 0.264	0.8558
Brain				
APC6 15 mg/kg	Vehicle control	−0.902	−1.276 to −0.528	<0.0001
AMPH-B 3 mg/kg	Vehicle control	−0.737	−1.111 to −0.363	<0.0001
MCFG 3 mg/kg	Vehicle control	−1.528	−1.902 to −1.154	<0.0001
APC6 15 mg/kg + MCFG 3 mg/kg	Vehicle control	−1.694	−2.068 to −1.319	<0.0001
APC6 15 mg/kg + MCFG 3 mg/kg	APC6 15 mg/kg	−0.791	−1.165 to −0.417	<0.0001
APC6 15 mg/kg + MCFG 3 mg/kg	AMPH-B 3 mg/kg	−0.957	−1.331 to −0.583	<0.0001
APC6 15 mg/kg + MCFG 3 mg/kg	MCFG 3 mg/kg	−0.166	−0.539 to 0.208	0.7224
MCFG 3 mg/kg	APC6 15 mg/kg	−0.625	−1.000 to −0.251	0.0002
MCFG 3 mg/kg	AMPH-B 3 mg/kg	−0.791	−1.165 to −0.417	<0.0001
APC6 15 mg/kg	AMPH-B 3 mg/kg	−0.166	−0.540 to 0.209	0.7231

To further validate the antifungal efficacy of APC6, we used histopathological analysis of tissues collected from AR 0384-infected mice. In four liver sections and two kidney and brain sections per mouse, we quantified fungal microabscesses, defined as aggregates of ≥10 yeast cells. In all three organs, the number of fungal microabscesses was significantly lower in the APC6-treated group than in the vehicle control group (Fig. S2).

### APC6 has no mutagenic activity in the Ames test

To evaluate the mutagenicity of APC6, we used *Salmonella* Typhimurium tester strains TA98 and TA100, both with and without metabolic activation (S9 mix). APC6 was cytotoxic at doses of ≥4.88 µg/plate in the absence of metabolic activation and at doses of ≥19.5 µg/plate in its presence. Across all concentrations tested, APC6 did not induce a twofold increase in the number of revertant colonies compared with the solvent control in either strain, or a dose-dependent increase (Table 3). These results suggest that APC6 is not mutagenic under the conditions tested.

**TABLE 3:** Evaluation of APC6 mutagenicity using the Ames test

	Compound	Dose/plate (μg)	Mean no. of revertant colonies/plate^*a*^	
			TA98	TA100
Without metabolic activation	APC6	0.153	19 ± 5.7	95 ± 2.1
		0.305	15 ± 0.7	97 ± 0.7
		0.610	14 ± 3.5	106 ± 4.2
		1.22	18 ± 3.5	101 ± 1.4
		2.44	15 ± 0.0	77 ± 1.4
		4.88	10 ± 2.1	63 ± 2.8
	DMSO^*b*^		18 ± 0.7	104 ± 2.8
	2-(2-Furyl)−3-(5-nitro-2-furyl)acrylamide	0.01	440 ± 32.5	684 ± 96.9
With metabolic activation	APC6	0.610	24 ± 1.4	109 ± 18.4
		1.22	26 ± 2.1	110 ± 0.7
		2.44	24 ± 3.5	112 ± 4.2
		4.88	26 ± 9.2	106 ± 14.1
		9.77	Toxic	19 ± 3.5
		19.5	Toxic	Toxic
	DMSO		28 ± 3.5	115 ± 9.9
	Benzo[a]pyrene	5	269 ± 11.3	1,338 ± 57.3

^*a*^ Mean ± standard deviation of duplicate determinations. Toxic, test compound is cytotoxic to the *Salmonella* test strain.

^*b*^ DMSO, dimethyl sulfoxide.

## DISCUSSION

To the best of our knowledge, this study is the first to demonstrate robust *in vivo* efficacy of the zinc-chelating compound APC6 (Fig. 1A) in a murine model of disseminated *C. auris* infection. APC6 significantly improved survival (Fig. 2) and reduced fungal burden in the liver, kidneys, and brain (Fig. 3; Fig. S2; Tables 1 and 2). These findings represent a critical advance, as no previous study has shown that zinc chelation can achieve such therapeutic effects against multidrug-resistant *C. auris* in a mammalian model. An initial evaluation *in vivo* suggested that APC6 (therapeutic dose, 15 mg/kg) had a favorable safety profile, with no substantial adverse effects during 28 consecutive days of administration (Fig. 1B and C). The ability of APC6 to improve survival and reduce fungal dissemination, together with its low toxicity and non-mutagenicity (Table 3), positions zinc chelation as a viable antifungal strategy. These findings support the concept that targeting fungal metal homeostasis offers a mechanistically distinct therapeutic approach.

Developing safe broad-spectrum antifungal agents remains extremely challenging (7). Although echinocandins are recommended by the CDC as first-line therapy for *C. auris* infections (https://www.cdc.gov/candida-auris/hcp/clinical-care/), resistance to this class of antifungals is emerging (23). A survey of *C. auris* isolates from Pakistan, India, South Africa, and Venezuela revealed resistance rates of 93% to fluconazole, 54% to voriconazole, 35% to amphotericin B, and 7% to echinocandins (5). Notably, 41% of the isolates were resistant to two antifungal classes, and 4% were resistant to all three major classes (5). As drug-resistant strains continue to spread, the risk of treatment-refractory infections is becoming an increasing clinical concern. In this context, our study provides *in vivo* evidence that zinc chelation can serve as a novel and effective therapeutic approach against multidrug-resistant *C. auris* infections. Importantly, APC6 was effective against two clinical isolates of *C. auris*, AR 0384 and AR 0389, which exhibit high-level resistance to fluconazole and reduced susceptibility to echinocandins or amphotericin B, respectively, indicating efficacy against *C. auris* strains with potentially distinct resistance mechanisms. As zinc plays a pivotal role in fungal virulence (18, 19, 24), including in enzymatic function, stress response, and biofilm formation, its chelation may exert antifungal effects through multifaceted disruption of fungal physiology.

Although APC6 did not surpass micafungin in reducing fungal burden in this study, its performance was similar, or superior, to that of amphotericin B (Fig. 3; Tables 1 and 2). AR 0384 exhibited high resistance to caspofungin (minimum inhibitory concentration = 16 µg/ml, Table S1) and the lowest susceptibility to micafungin (2 µg/mL) among the CDC reference strains, which remains below the tentative resistance breakpoint (≥4 µg/mL), and responded to micafungin treatment in our model. However, highly micafungin-resistant *C. auris* isolates have been reported in clinical settings (25), underscoring the need for alternative therapeutic strategies. Moreover, several clinically important fungal pathogens, including species of *Fusarium*, *Trichosporon*, and members of the *Mucorales* order, are intrinsically resistant to echinocandins (26). As APC6 targets a mechanism distinct from those targeted by existing antifungal agents, it may also be efficient against such pathogens. Our future studies will aim to expand the evaluation of APC6 across a broader range of clinically relevant species.

The findings of this study, along with previous research (27), suggest that targeting fungal metal homeostasis represents a viable novel strategy. *PRA1* and *ZRT1* in *C. albicans*, as well as their orthologous genes in other *Candida* spp., encode zincophores that bind zinc with high affinity and function as endogenous zinc-capturing molecules facilitating zinc uptake under limiting conditions (28). In the presence of zinc-chelating compounds such as APC6 and 19ak, intracellular zinc levels in fungal cells decrease, accompanied by marked upregulation of zincophore-related genes (18, 24). This indicates that APC6 induces zinc starvation by outcompeting the endogenous zincophores and may have an even higher zinc-binding affinity. In addition to zinc, there has been considerable interest in other essential trace metals, such as iron and copper, as potential antifungal targets. Iron chelators such as deferiprone and deferasirox, originally developed for the treatment of iron overload in patients requiring chronic blood transfusions, have demonstrated encouraging results in both murine (29–31) and human (32–34) models of fungal infections, including candidiasis, mucormycosis, and aspergillosis.

The safety profile of APC6 at the therapeutic dose was favorable under the conditions tested, as no mortality, significant clinical abnormalities, or major organ toxicity was observed during the 28-day repeated-dose study (Fig. 1B and C). However, determination of the maximum tolerated dose revealed that APC6 has a narrow margin between the effective and toxic doses, with lethality observed at 45 mg/kg. This contrasts with conventional antifungal agents such as echinocandins or azoles, which typically have broader safety margins (35). Therefore, further optimization of APC6 dosing strategies or testing of its derivatives will be essential. In the Ames test, APC6 had no mutagenic activity in either the TA98 or the TA100 strain, with or without metabolic activation (Table 3). These results suggest a low risk of genotoxicity at therapeutically relevant concentrations, although more comprehensive genotoxicity testing, including mammalian cell assays, will be required in future preclinical evaluations.

Although APC6 has shown promising *in vivo* effectiveness, several challenges remain in its development as a drug candidate. One major limitation, as noted earlier, is the narrow range between therapeutic benefit and toxicity. Given that zinc interacts with a wide range of biomolecules (11), chelating it could lead to off-target effects in host cells, raising concerns about APC6 safety. While the toxicity of APC6 to human cells was low in our *in vitro* study (18), structural modifications are necessary to improve its safety profile for potential clinical applications. In this study, we used intraperitoneal administration of APC6, whereas future clinical development will require evaluation of its oral or intravenous administration, along with comprehensive pharmacokinetic and pharmacodynamic assessments. Data on tissue distribution and the feasibility of systemic formulations will be essential to support future clinical use. Finally, in this study, APC6 was administered starting 24 h before *C. auris* infection, as in a previous study (19). Additional *in vivo* studies will be necessary to fully establish the clinical applicability of APC6, including delayed treatment models, in which APC6 is administered after infection onset.

In conclusion, APC6 was efficient in a murine model of multidrug-resistant *C. auris* infection, supporting its utility as a novel antifungal agent. This study establishes zinc-chelating compounds as a new class of antifungals and highlights their promise in overcoming resistance to existing antifungal therapies. Our findings provide valuable information for the development of future antifungal agents with improved potency and safety profiles.

## MATERIALS AND METHODS

### Mice

Six- to seven-week-old female C57BL/6J mice were purchased from Japan SLC, Inc. (Shizuoka, Japan) and maintained under specific pathogen-free conditions at the National Institute of Infectious Diseases in Japan.

### *In vivo* candidiasis model

A murine model of systemic candidiasis was established according to a previously described method (19). Mice were intraperitoneally injected with cyclophosphamide (200 mg/kg body weight; Shionogi, Osaka, Japan) on day −1 and were provided with drinking water supplemented with 50 ppm enrofloxacin (LKT Labs, Saint Paul, MN, USA) to prevent bacterial infections (36). *Candida auris* was cultured overnight at 35°C in yeast extract–peptone–dextrose (YPD) (1% Bacto Yeast Extract [Difco, Detroit, MI, USA], 2% Bacto Peptone [Difco], and 2% glucose; pH 6.8) broth. On day 0, mice were intravenously infected with a 0.1 mL *C*. *auris* inoculum containing either 5 × 10⁷ CFU/mouse (strain AR 0384) or 3 × 10⁷ CFU/mouse (strain AR 0389). Beginning on day −1, APC6 was injected intraperitoneally once daily at doses of 5 or 15 mg/kg; amphotericin B or micafungin (both at 3 mg/kg; Tokyo Chemical Industry Co., Ltd., Tokyo, Japan) was injected intraperitoneally once daily. In the zinc supplementation group, ZnSO₄ (9.0 mg/kg, corresponding to a 1.5-fold molar excess of APC6) was administered intraperitoneally 20 minutes after APC6. All drug solutions were filtered through 0.22 µm polyvinylidene difluoride filters.

### Survival analysis

Survival was monitored for 7 days post-inoculation. Data were collected from at least three independent experiments. Differences from vehicle control were analyzed by the log-rank test.

### Determination of organ fungal burden

To evaluate the therapeutic efficacy of each treatment, the livers, kidneys, and brains were collected on day 3, weighed, and homogenized in PBS. The homogenates were serially diluted and plated on YPD agar plates supplemented with 1% penicillin–streptomycin to inhibit bacterial growth. Plates were incubated at 35°C for 24 h, and fungal burden per organ was determined by counting CFU.

### Histopathological analysis

Organs were excised, fixed in 10% neutral-buffered formalin, dehydrated with ethanol, and embedded in paraffin by following standard procedures. Tissue sections were mounted onto glass slides and stained with hematoxylin and eosin and with Grocott–Gomori’s methenamine silver. Slides were examined by light microscopy.

### Toxicity evaluation in mice

In the single-dose de-escalation study, mice received a single intraperitoneal injection of APC6 at doses of 45, 30, or 15 mg/kg, or vehicle control and were observed for 2 days. During this period, body weight and activity levels were monitored. Activity was graded on a 5-point scale (37), as follows: grade 1 (very active): highly curious, strong and rapid movement, with normal food intake; grade 2 (active): strong and rapid movement with occasional pauses, normal food intake; grade 3 (less active): appropriate response to stimulation but frequent interruptions in activity, with reduced food intake; grade 4 (slow): reduced activity with slow movements and markedly decreased food intake; grade 5 (lethargic): immobile with no food intake. In the 28-day repeated-dose toxicity study, mice (*n* = 12 per group) were injected intraperitoneally with APC6 at 15 mg/kg once daily for 28 consecutive days. Control mice received vehicle only. Activity levels were monitored daily at the time of APC6 or vehicle injection, and body weight was recorded on days 0 and 28 (immediately before necropsy).

### Ames test

The assay was performed by BoZo Research Center Inc. (Tokyo, Japan). The mutagenicity of APC6 was evaluated using the plate incorporation method with *S.* Typhimurium tester strains TA98 and TA100 (38). The test was conducted either with or without metabolic activation using a rat liver S9 fraction (10% [vol/vol]) from rats pretreated with phenobarbital and 5,6-benzoflavone. Serial dilutions of APC6 were tested at concentrations ranging from 1.22 to 5,000 µg/plate (each dilution 100 µL). As a positive control, 2-(2-furyl)−3-(5-nitro-2-furyl)acrylamide was used in the absence of metabolic activation and benzo[a]pyrene was used under metabolic activation. Dimethyl sulfoxide was used as a vehicle control. The number of revertant colonies and the condition of the background bacterial lawn were assessed by comparison with the control. APC6 was considered mutagenic if the mean number of revertant colonies exceeded twice that in the control and was dose dependent.

### Statistical analyses

Statistical analyses were performed in GraphPad Prism, version 8 (GraphPad Software, La Jolla, CA, USA). One-way analysis of variance with Tukey’s multiple comparison post-test was used to analyze more than two groups. For fungal microabscesses data, the Kruskal–Wallis test, followed by Dunn’s multiple comparison test, was applied. *P* values of <0.05 were considered statistically significant in all tests.

## Data Availability

Data are available in the supplemental material.

## SUPPLEMENTAL MATERIAL

Supplemental figures aac.00856-25-s0001.docx Fig. S1 and S2.

Table S1 aac.00856-25-s0002.xlsx MIC values of existing antifungal agents against *C. auris*.

ASM does not own the copyrights to Supplemental Material that may be linked to, or accessed through, an article. The authors have granted ASM a non-exclusive, world-wide license to publish the Supplemental Material files. Please contact the corresponding author directly for reuse.
