# Supplementary material for: A zinc-chelating cyclic alkyl polyamine compound is efficient and safe in a murine model of multidrug-resistant Candida auris infection
Source: Antimicrob Agents Chemother. 2025 Sep 26;69(11):e00856-25. doi: 10.1128/aac.00856-25 (PMC12587541; doi:10.1128/aac.00856-25)
Supplement: Supplemental figures — Fig. S1 and S2. [file aac.00856-25-s0001.docx]

**Supplemental Information**

**A zinc-chelating cyclic alkyl polyamine compound is efficient and safe in a murine model of multidrug-resistant *Candida auris* infection**

Takayuki Shinohara^a^, Akira Wada^b^, Masahiro Abe^a^, Sayoko Oiki^a^, Ami Koizumi^a^, Amato Otani^a^, Harutaka Katano^c^, and Yoshitsugu Miyazaki^a,d #^

^a^ Department of Fungal Infection, National Institute of Infectious Diseases, Japan Institute for Health Security, Tokyo 162-8640, Japan

^b^ Center for Integrative Medical Sciences, RIKEN, Kanagawa 230-0045, Japan

^c^ Department of Infectious Disease Pathology, National Institute of Infectious Diseases, Japan Institute for Health Security, Tokyo 162-8640, Japan

^d^ Leprosy Research Center, National Institute of Infectious Diseases, Japan Institute for Health Security, Tokyo 189-0002, Japan

^#^ Corresponding author:

Yoshitsugu Miyazaki, MD, PhD

Address: 1-23-1 Toyama, Shinjuku-ku, Tokyo 162-8640, Japan

Fax +81-3-5285-1272

Tel +81-3-5285-1111

Email ym46@niid.go.jp

**Supplemental Figures**

**
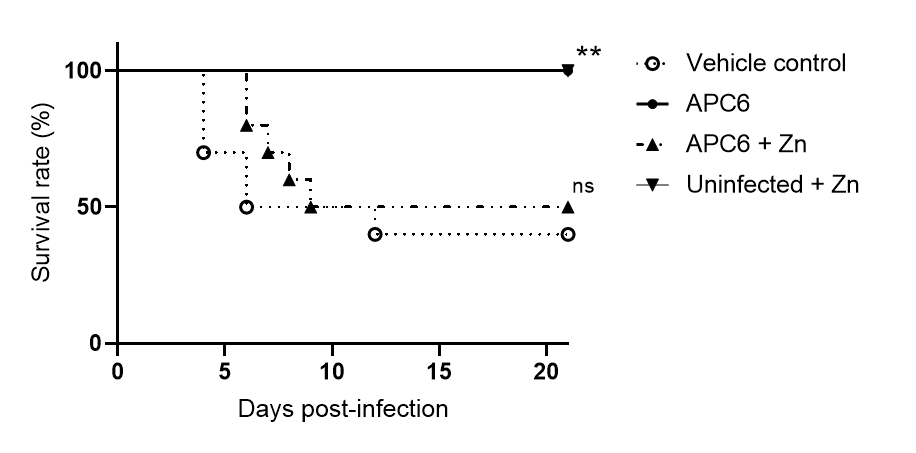
**

**FIG S1** Kaplan–Meier survival curves of mice infected with *Candida auris* AR 0384 and treated with APC6 with or without zinc supplementation. Mice (n = 10 per group) were injected intraperitoneally with APC6 (15 mg/kg), or ZnSO₄ (9.0 mg/kg; 1.5-fold molar excess), or both. Survival was monitored for 21 days post-infection. Statistical significance was assessed by the log-rank test. ***P* < 0.01; ns, not significant (vs. vehicle control).


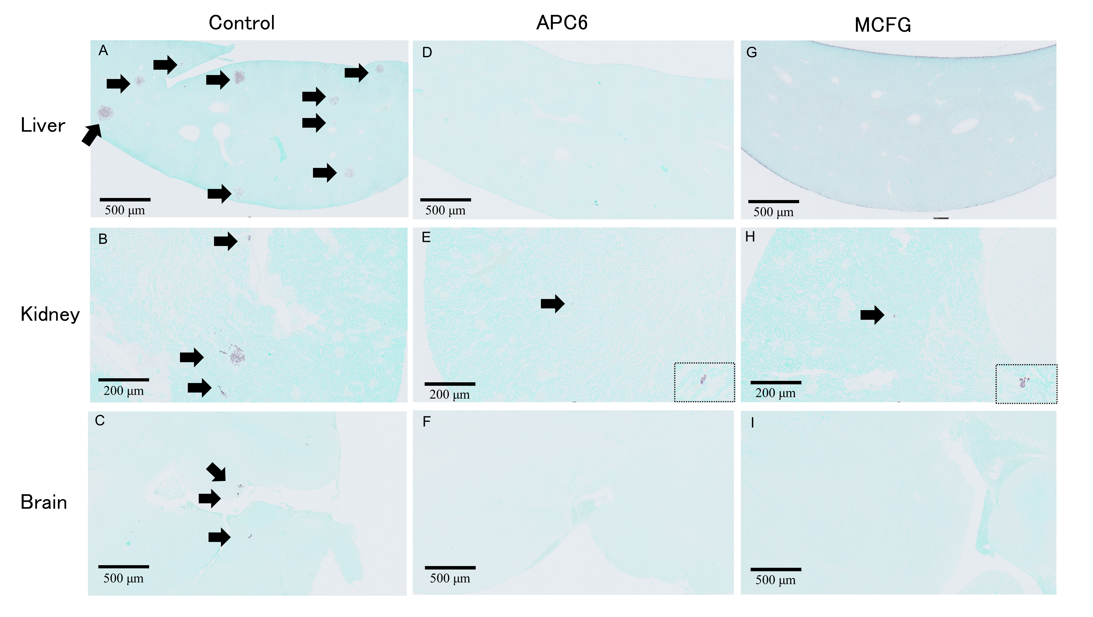


J

**
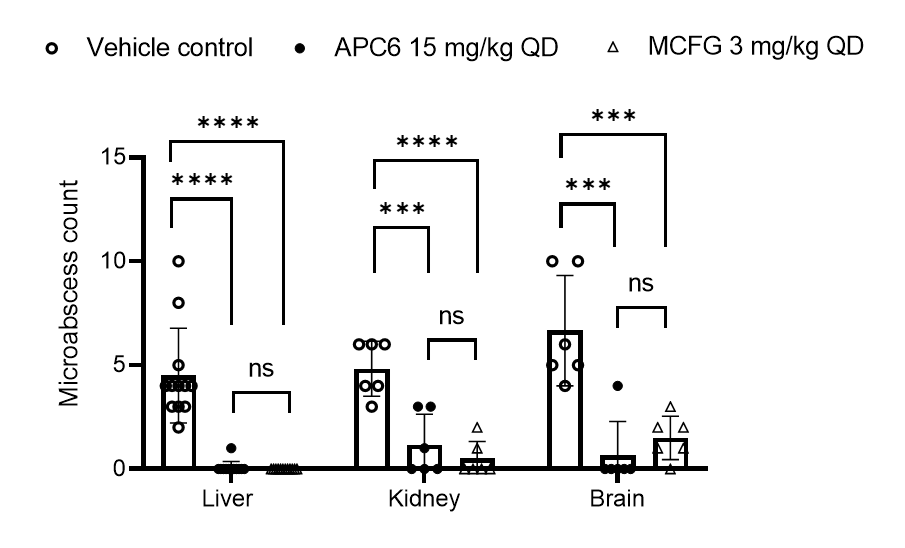
**

**FIG S2** Representative histopathological sections of formalin-fixed, paraffin-embedded liver, kidney, and brain tissues collected on day 3 post-infection with *Candida auris* AR 0384. (A, D, G) Liver sections. (B, E, H) Kidney sections. (C, F, I) Brain sections. (A–C) Vehicle control group. (D–F) APC6-treated group. (G–I) Micafungin (MCFG)-treated group. Sections were stained with Grocott–Gomori’s methenamine silver stain to visualize yeast cells. Arrows indicate fungal microabscesses. (J) Quantification of fungal microabscesses in each group. Three mice per group were evaluated by using four liver and two kidney and two brain sections per mouse. *** *P* < 0.001; **** *P* < 0.0001; ns, no significant difference (Kruskal–Wallis test followed by Dunn’s multiple comparison test). QD, once daily
